# Characterization of the complete chloroplast genome of a chinese endangered species *Cymbidium wenshanense* Y. S. Wu et F. Y. Liu

**DOI:** 10.1080/23802359.2023.2241695

**Published:** 2023-08-03

**Authors:** Xiaomeng Wang, Longjie Cheng, Zhilin Li, Zichen Zhang, Yuying Wang

**Affiliations:** College of Horticulture and Landscape, Yunnan Agricultural University, Kunming, China

**Keywords:** *cymbidium wenshanense*, chloroplast genome, phylogenetic analysis

## Abstract

*Cymbidium wenshanense* Y. S. Wu et F. Y. Liu is of significant ornamental and breeding value. In this study, Illumina high-throughput sequencing was used to sequence and analyze the complete chloroplast genome of *C. wenshanense*, and the phylogenetic relationships between this and other Orchidaceae species were established. The whole chloroplast genome was 156,292 bp in length and contained 84 mRNA genes, 45 tRNA genes, and 4 rRNA genes. The phylogenetic tree of 11 Orchidaceae species revealed *C. wenshanense* to be most closely related to *Cymbidium mastersii*.

## 1. Introduction

**Figure 1.**
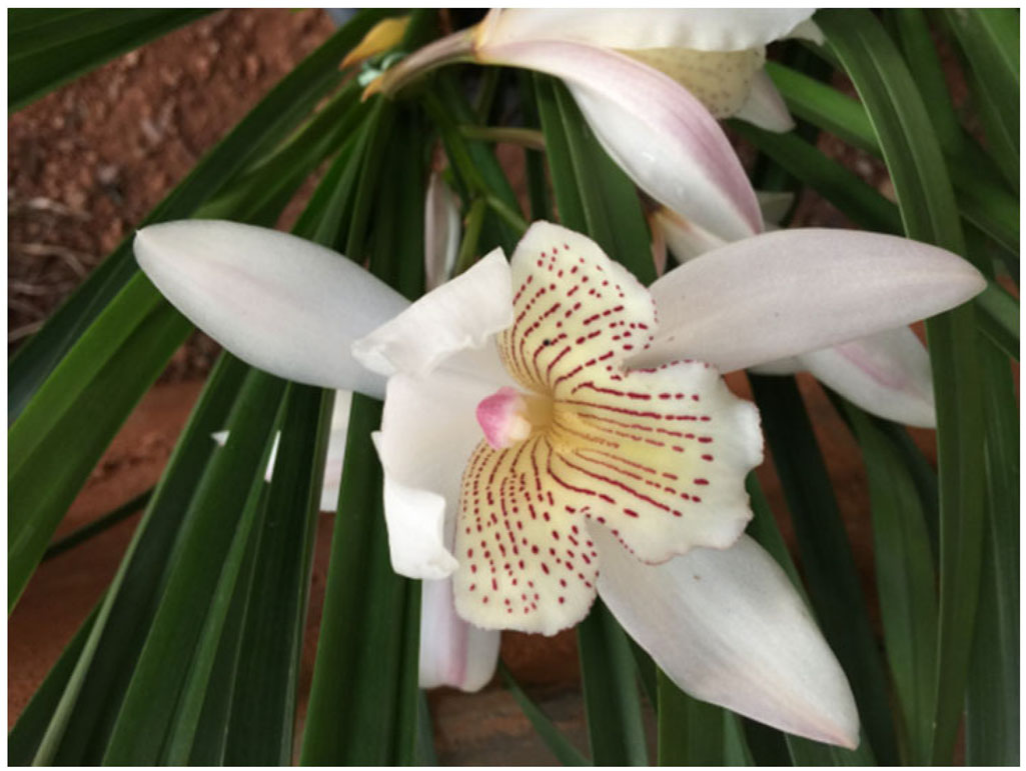
*Cymbidium wenshanense* ‘fenji’. Photograph by Xiaomeng Wang on Mar 15, 2022.

*Cymbidium wenshanense* (Wu & Liu 1990) is a member of the Orchidaceae and was discovered in 1990 in Wenshan, Yunnan Province, China (Wu and Liu 1990). *C. wenshanense* is a perennial epiphytic herb of the subgenus *Cymbidium*, mainly distributed in southeastern Yunnan and northern Vietnam (Chen and Ji 1998). The breed used in this article was *Cymbidium wenshanense ‘*fenji’, which was selected and bred by the College of Horticulture and Landscape, Yunnan Agricultural University, and obtained a ‘new variety’ certificate for Yunnan Province registration in 2015. This species is distributed in the southeast of Yunnan, between 1000 meters and 1800 meters above sea level, and its habitat consists of forest trees. Because it is a potted variety selected by us, we did not go deep into the field to collect it. The leaves of *C. wenshanense* ‘fenji’ are banded, dark green, slightly yellow, thick, and glossy. Each inflorescence slants outward, harboring 3–10 flowers that are 6–9 cm in diameter. The sepals are light brown on the underside, and the buds are pink. The petals are white, with indistinct red-brown veins, and the lip is white, with red-brown veins and spots. An elegant fragrance is given off during the whole flowering period, from January to March (Ding et al. 2007). In this study, we describe a new chloroplast genome that is different from those previously published for *C. wenshanense*, which will aid investigations into the diversity and genetic resources of this plant and provide a theoretical basis for breeding new *C. wenshanense* varieties. Figure 1 shows a reference image taken by Xiaomeng Wang on Mar 15, 2022.

## 2. Materials and methods

### 2.1. Sample collection and preservation

Resources of this plant are stored in the flower Institute of the Wildlife Germplasm Resources Bank of Southwest China at Yunnan Agricultural University (25.1319° N, 102.74912° E). Specimens of *C. wenshanense* ‘fenji’ were gathered from the College of Horticulture and Landscape, Yunnan Academy of Agricultural University (25.1319° N, 102.74912° E) by Yuying Wang. A specimen was deposited at the Flower Institute of Yunnan Agricultural University (25.1319° N, 102.74912° E) (Yuying Wang, wyysxp@126.com) under the voucher number 000030. A CTAB method (Doyle and Doyle 1987) was used to extract the entire chloroplast DNA from *C. wenshanense* using fresh mesophyll tissue.

### 2.2. DNA extraction, sequencing, and assembly

The tissue sample used for sequencing was kept with the corresponding voucher samples. Sequencing was performed using the Illumina NovaSeq system at GENOSEQ Technologies Ltd. (Wuhan, China). Genomic DNA was extracted using the CTAB method and tested for quality. The DNA was then segmented by mechanical interruption (ultrasonic) and purified. DNA end repair was carried out and a single 3′ A residue was added before analysis using the sequence-tag connector approach. Agarose gel electrophoresis was used to select the segment size, and PCR amplification was performed to create a sequencing library (NEBNext UltraDNA Library Prep Kit for Illumina). The library was then subjected to quality inspection, and the qualified library was sequenced using an Illumina NovaSeq system. The quality check was conducted in SOAPnuke (version:1.3.0), with the following conditions: (1) reads with >5% N base content were removed; (2) low quality reads (mass value ≤5) were removed when the number of low quality bases exceeded 50%; and (3) reads contaminated with adapters were removed. The raw and clean reads were obtained and assembled with GetOrganelle v1.7.1 (Jian et al. 2020).

### 2.3. Annotation and analysis

The assembled contigs were then compared with the chloroplast genomes of closely related species using a BLASTN search (BLAST 2.2.30þ; E value 1 × 10^−5^). Then, the contigs were checked, selected and adjusted to obtain the final data set. The chloroplast genome was annotated and mapped using the Plastid Genome Annotator (Qu et al. 2019).

## 3. Results

### 3.1. Genomic characterization

**Figure 2.**
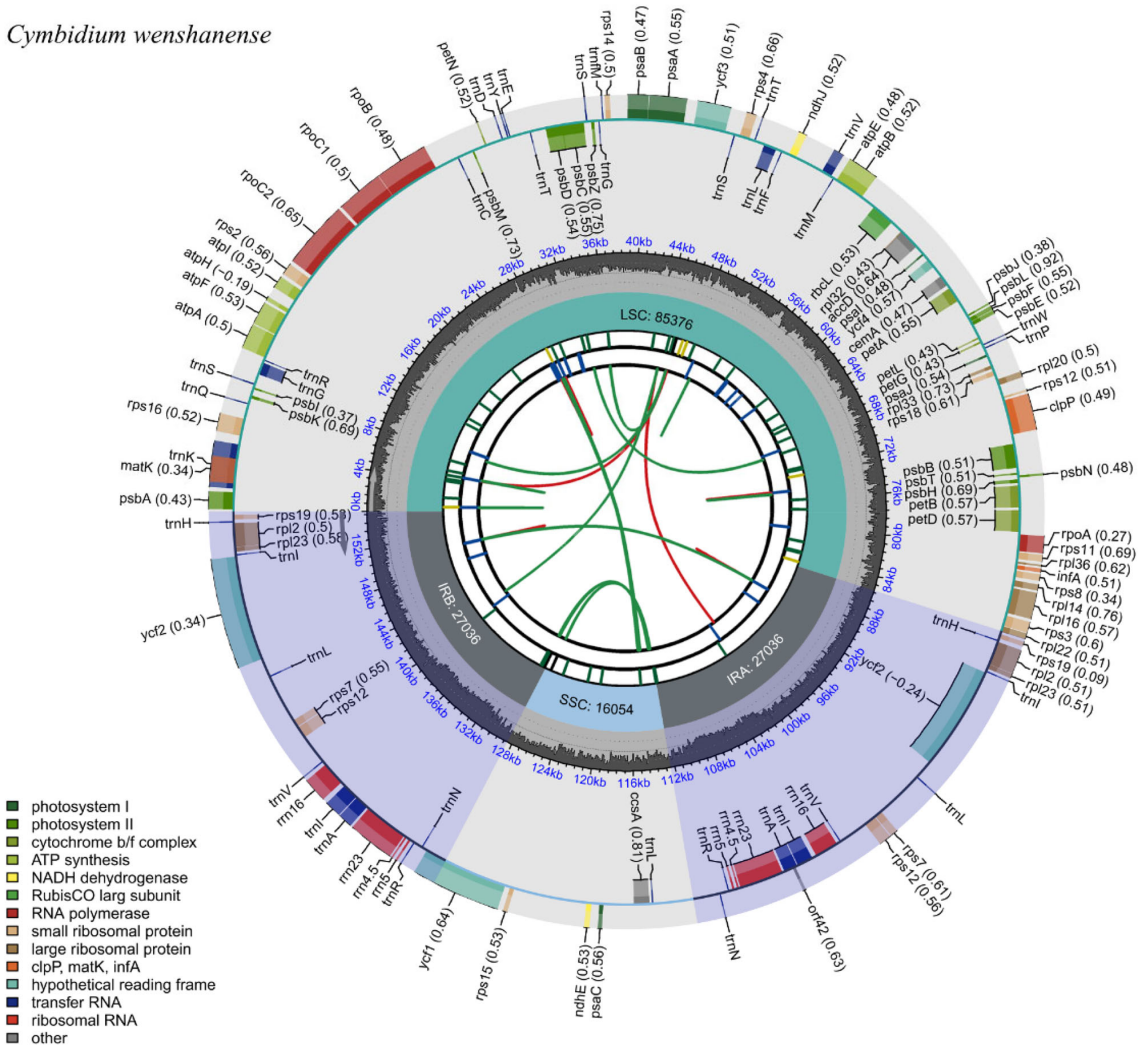
The chloroplast genome map of *Cymbidium wenshanense*. Genes drawn outside the outer circle were transcribed in a counterclockwise direction, and genes drawn inside the outer circle were transcribed clockwise. Genes that belong to different functional groups are color-coded. The colored legend at the bottom indicates genes with different functions. The dark grey inner circle indicates the GC content of the chloroplast genome and the presence of nodes in the LSC, SSC, and IR regions.

The length of the complete chloroplast genome of *C. wenshanense* was found to be 155,502 bp. The genome presented a characteristic quadripartite circular structure, which included one pair of inverted repeat (IR) regions (27,036 bp), one large single-copy (LSC) region (85,376 bp), and one small single-copy (SSC) region (16,054 bp). Furthermore, the complete genome contained 77 mRNA genes, 38 tRNA genes, and 8 rRNA genes. The overall GC content of the *C. wenshanense* chloroplast genome was 36.8%, and the GC contents of the IR, LSC, and SSC regions were 42.8%, 34.2%, and 30.7%, respectively. The chloroplast genome map of *Cymbidium wenshanense* is shown in Figure 2.

Apart from ON782579, we found another complete *C. lancifolium* chloroplast genome, which has been deposited in GenBank (MK848057). According to the BLAST results, the per. ident of MK848057 was 99.47% and the score was 50503. The length of the MK848057 genome was 157,740 bp, and it included one pair of IR regions (26,073 bp), one LSC region (85,333 bp), and one SSC region (18,879 bp). Furthermore, the complete genome contained 77 mRNA genes, 36 tRNA genes, and 8 rRNA genes. The overall GC content of the genome was 29.1%, and the GC contents of the IR, LSC, and SSC regions were 43.3%, 34.2%, and 29.1%, respectively.

### 3.2. Phylogenetic analysis

**Figure 3.**
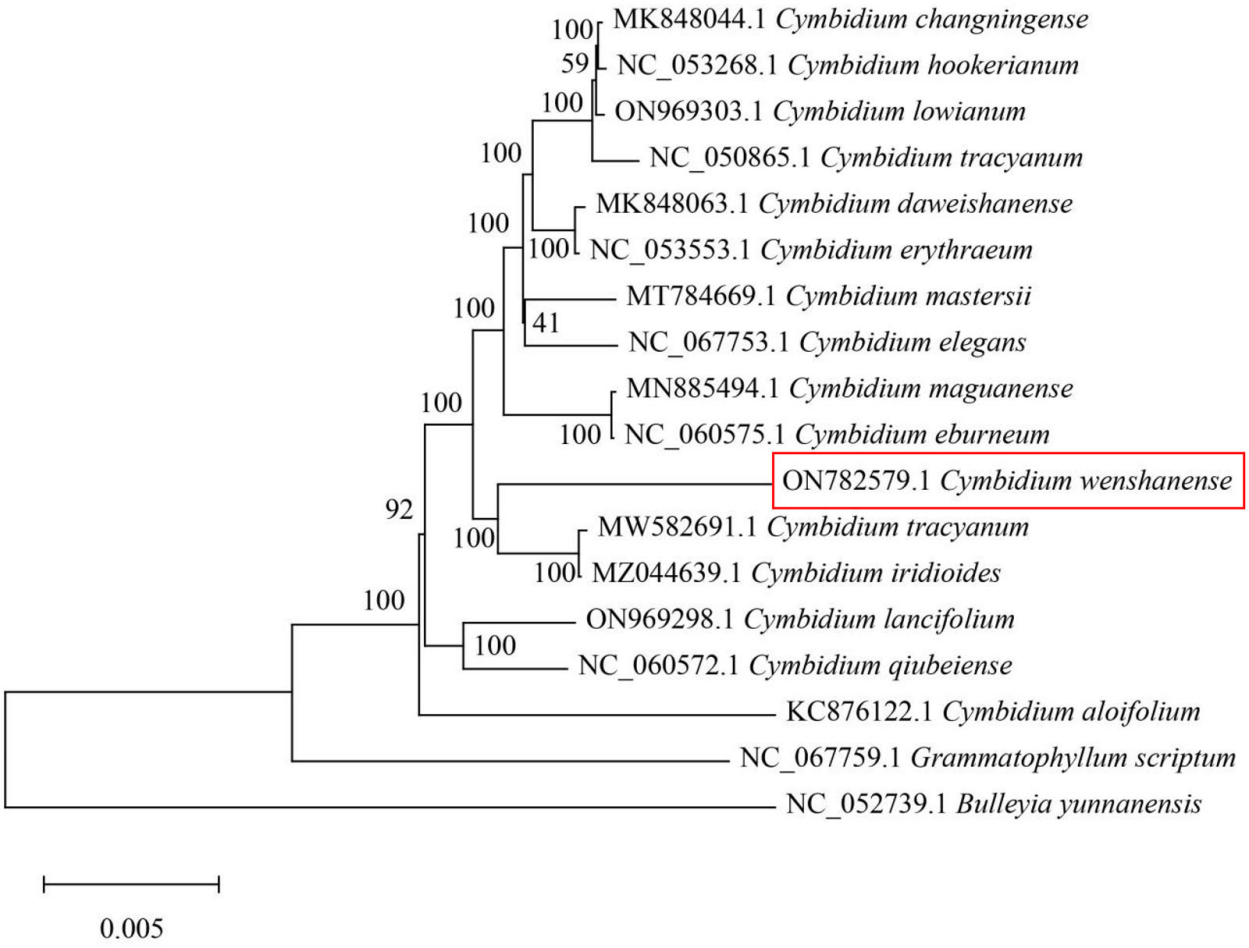
Phylogenetic tree based on 18 complete chloroplast genome sequences from Orchidaceae species. The following sequences were used: *Cymbidium changningense* (MK848044; Zheng et al. 2019), *Cymbidium hookerianum* (NC_053268), *Cymbidium lowianum* (ON969303), *Cymbidium tracyanum* (NC_050865), *Cymbidium daweishanense* (MK848063), *Cymbidium erythraeum* (NC_053553), *Cymbidium mastersii* (MT784669), *Cymbidium elegans* (NC_067753), *Cymbidium maguanense* (MN885494; Zheng et al. 2020), *Cymbidium eburneum* (NC_060575), *Cymbidium tracyanum* (MW582691; Zhe et al. 2022), *Cymbidium iridioides* (MZ044639), *Cymbidium lancifolium* (ON969298), *Cymbidium qiubeiense* (NC_060572.1), *Cymbidium aloifolium* (KC876122; Yang et al. 2013), *Grammatophyllum scriptum* (NC_067759), and *Bulleyia yunnanensis* (NC_052739).

To study the phylogenetic relationships of *C. wenshanense* with other species, a phylogenetic tree was constructed using 15 complete chloroplast genomes from *Cymbidium* species. Two other Orchidaceae species were selected as an outgroup. All sequences were downloaded from the NCBI GenBank and aligned using the online program MAFFT v7. MEGA v7.0 was used to build a maximum-likelihood phylogenetic tree with 1000 rapid bootstrap replicates (Kumar et al. 2016). The phylogenetic tree analysis indicated that *C. wenshanense* was closely related to *Cymbidium tracyanum* (Figure 3).

## 4. Discussion and conclusion

The complete chloroplast genome of *Cymbidium wenshanense* ‘fenji’ was sequenced on the Illumina NovaSeq system, and the length was found to be 155,502 bp (Genbank accession no. ON782579). The phylogenetic position of *C. wenshanense* ‘fenji’ within the subfamily of Orchidaceae was determined, and the results showed that *C. wenshanense* ‘fenji’ was closely related to *C. tracyanum*. The chloroplast genomic data of *C. wenshanense* ‘fenji’ will be essential for further genetic studies of evolution, taxonomy, DNA barcoding, resource conservation, and phylogenetic research.

## Data Availability

The data that support the findings of this study are openly available from the NCBI GenBank at https://www.ncbi.nlm.nih.gov, reference number ON782579. The associated BioProject, SRA and Bio-Sample numbers are PRJNA764364, SRR15959874 and SAMN21500377, respectively.
